# Where Have All the Giants Gone? How Animals Deal with the Problem of Size

**DOI:** 10.1371/journal.pbio.2000473

**Published:** 2017-01-11

**Authors:** Taylor J. M. Dick, Christofer J. Clemente

**Affiliations:** 1 Department of Biomedical Physiology and Kinesiology, Simon Fraser University, Burnaby, British Columbia, Canada; 2 School of Science and Engineering, University of the Sunshine Coast, Sippy Downs, Queensland, Australia; Lund University, Sweden

## Abstract

The survival of both the hunter and the hunted often comes down to speed. Yet how fast an animal can run is intricately linked to its size, such that the fastest animals are not the biggest nor the smallest. The ability to maintain high speeds is dependent on the body’s capacity to withstand the high stresses involved with locomotion. Yet even when standing still, scaling principles would suggest that the mechanical stress an animal feels will increase in greater demand than its body can support. So if big animals want to be fast, they must find solutions to overcome these high stresses. This article explores the ways in which extant animals mitigate size-related increases in musculoskeletal stress in an effort to help understand where all the giants have gone.

## Introduction

The largest animals to have ever walked the earth have long gone extinct. Fossil evidence suggests that the largest of these animals, dinosaurs, reached masses of over 50,000 kg [1]. Given their enormous mass, it remains a mystery as to how they could have walked, and yet the only complete evidence that we have for how animal locomotion changes with body size lies within living species. Being big has advantages—large animals have fewer predators [2] and more reserves available [3], are better at retaining body heat [4], and are able to travel farther distances owing to their lower relative cost of transport [5]. However there is also a major disadvantage to being big. The body mass of an animal is proportional to the gravitational force that it experiences. So as an animal increases in mass, the amount of force that its skeleton needs to support or resist must also increase. This would not be a problem if an animal’s ability to support this mass also increased equally, but this is not the case. Furthermore, the performance of an animal, such as the speed at which it can run, is linked to this mismatch between body size and the reduced ability to support this mass.

Being big has advantages, but so does being fast [6,7]. In order to deal with the problem of size, different groups of animals have adopted a variety of strategies that act to reduce musculoskeletal stress. However, which stress reduction strategies are exploited may influence the relationship between an animal’s speed and its size, constraining how fast an animal can run. Being large and slow is potentially maladaptive and may have contributed to downfall of these (now extinct) giants. But were they large and slow? In order to understand the solutions to reduce mechanical stress and the size limitations to speed, we must first understand what causes this increase in stress as animals get big.

**Fig 1:**
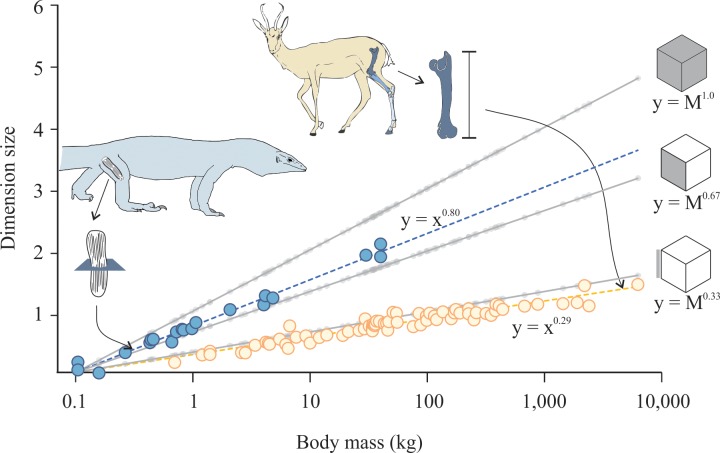
Scaling of length, area, and volume dimensions with body mass. Body masses for all mammals and lizards (data from [10–12]) are shown with predictions based on geometric similarity for length, area, and volume. Regression lines are shown for geometric scaling (isometry) for length, area, and volume (grey lines). Scaling relationships are also shown for muscle area (physiological cross-sectional area) in the iliofibularis of varanids (dashed blue line), which scales with positive allometry (original data reported in [12]) and femur length in mammals (dashed yellow line), which scales with negative allometry (original data reported in [10,11]).

If animals were to increase in size but keep the same shape, i.e., scale geometrically (or isometrically), their linear dimensions would increase proportional to body mass (M)^0.33^ and area dimensions would increase proportional to M^0.67^ (Fig 1). Stress depends on the amount of force applied over a certain cross-sectional area (*σ* = *F/A*), and because force increases proportional to M^1.0^ and area increases proportional to M^0.67^, geometric scaling predicts that stresses placed on bones and muscles will increase with M^0.33^. So as animals increase in size, there is a force mismatch—the changes in limb dimensions cannot keep up with the increased mechanical demands of support. Yet there are still some living examples of large animals in nature, so the question arises: How do they deal with the size-related increases in stress? Do these large animals simply operate dangerously close to bone and muscle failure points? Studies in extant animals have suggested that this is probably not the case. Animals ranging from 0.04 to 300 kg maintain similar safety factors, operating with peak stresses during locomotion that are two to ten times lower than their failure stress [8,9]. In this article, we describe five possible solutions by which animals could avoid the mechanical consequences of size, and this might give us insight into how fast these giants could have moved. Animals could get bigger either by (1) modifying the material properties of their bones and muscles, (2) changing the geometric shape of their bones, (3) changing the way their bones are loaded, (4) modifying the architecture of their skeletal muscles, or (5) suffering decreases in locomotor performance and simply slowing down.

## Bone (or Muscle) Itself Gets Stronger

Large animals could deal with the size-predicted increases in stress by evolving relatively stronger bones or muscles. Yet early studies measured the stresses under which bones fail and reported no difference in the failure stress of bones in animals ranging from less than 0.04 to nearly 700 kg [13,14]. Like bone, the properties of skeletal muscle are highly conserved. In fact, the arrangement of skeletal muscles’ molecular motor proteins actin and myosin does not vary across taxa [15]. Furthermore, Seow and Ford [16] measured the mechanical properties of skeletal muscle across a 25,000-fold size range and found no difference in fibre diameter or maximum isometric force, suggesting that the maximum stress within muscle of both small and large animals is constant [15,17]. However, a recent modelling study suggests that bone trabecular architecture from both mammalian and avian species ranging from 0.003 to 3,400 kg does vary with body size [18]. Although this may reduce bone strain at the cellular level, it remains unclear whether this can mitigate size-related increases in stress at the skeletal level, so other solutions may be necessary.

## Bones Get Thicker

Another possible mechanism to deal with increased stress is to change the shape of bones. Evolving increasingly thicker or increasingly shorter limb bones would reduce the magnitude of bone stress. McMahon [19,20] proposed a scaling model termed “elastic similarity” in which animals optimize the shape of their bones to become more stout and better able to withstand increased forces. This model has been shown to agree well with ungulate limb bones [21,22] and in the humerus of both reptiles and mammals [23], but other studies show that when a large size range of mammals is considered, bone dimensions scale closer to isometry [10,11,24,25]. Studies have also estimated the scaling of bone dimensions in extinct taxa from fossil evidence and revealed general similarities between dinosaurs and mammals—with bones becoming slightly thicker in larger species as compared to smaller ones [26,27]. Thus, it appears that the scaling of limb bone geometry exhibits a large amount of variation, and there is likely not a universal scaling theory available to describe all terrestrial vertebrates [23].

However, even if bones scaled according to McMahon’s elastic theory, this still may not be enough. Biewener [13] has shown that even with these slightly thicker bones, the peak stresses would still increase in mammals with M^0.28^. Other groups of animals show a similar pattern. Among varanid lizards, for example, limb bone diameters scale with positive allometry and, similar to mammals, comparisons with theoretical models show that these size-related changes in limb bone geometry are unable to fully compensate for size-related increases in limb bone stress [28]. Thus, it appears that changes in bone properties or changes in bone shape do not fully counteract the size-related increases in stress. Possibly, large animals could compensate by decreasing the mechanical forces acting on their musculoskeletal system.

## Posture Changes

**Fig 2:**
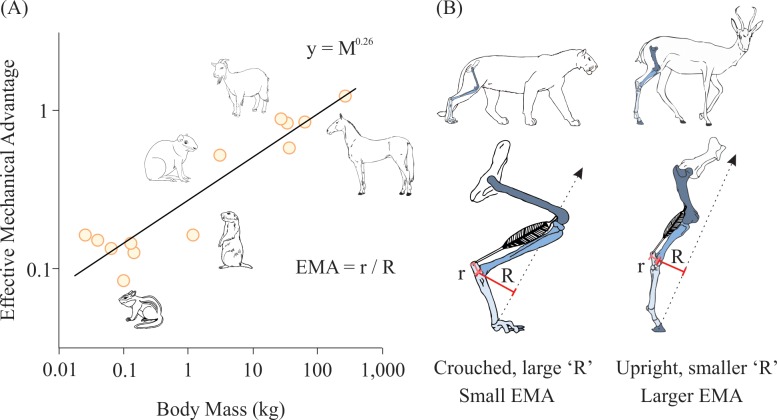
Effect of body size and posture on limb EMA. (A) Hindlimb EMA scaling for mammals (data reported in [17, 31]) shows that EMA scales with M^0.26^. (B) Effect of hindlimb posture on limb EMA (ratio of extensor muscle moment arm (r) to the resultant three-dimensional ground reaction force moment arm (R) shows that crouched animals have a lower limb EMA than upright animals. Dashed black arrows represent the ground reaction force vector.

For centuries, scientists have observed that small mammals typically move with crouched limb postures in which their limbs are flexed underneath their body, whereas larger animals move with an upright posture and extended joints [29–31]. By adopting a more upright posture, large animals are able to increase the effective mechanical advantage (EMA) of their limb joints (Fig 2) and consequently decrease musculoskeletal stress during locomotion [31,32]. EMA has been shown to scale with M^0.25^, which nearly matches the predicted increase in stress that would occur if posture did not change (Fig 2) [17,31]. However there is likely an upper limit to EMA in large terrestrial animals—at body masses greater than ~300 kg, EMA no longer increases linearly with body mass because an animals limbs are nearly as extended as possible [17,32,33]. Thus, posture seems like a plausible mechanism for why bone and muscle stresses remain unchanged among phylogenetically diverse groups of mammals [17,32] and birds [34], at least at masses less than 300 kg. But even among these smaller size ranges, this stress reduction strategy may not be ubiquitously employed across all taxa.

**Fig 3:**
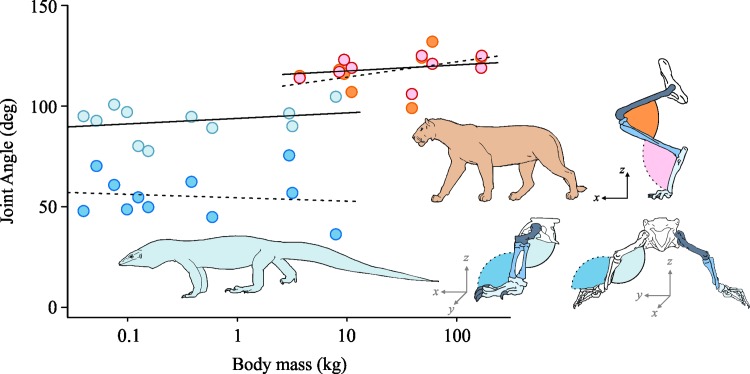
Postural changes with body size in felids and varanids. Joint angles for felids (ankle: red points, dashed line; knee: orange points, solid line) and varanids (ankle: dark blue points, dashed line; knee: light blue points, solid line) display that posture remains relatively unchanged across body masses in both groups and that varanids adopt a more crouched posture compared to felids. Joint angles for felids are represented in a two-dimensional sagittal plane, whereas for varanids, angles are given as the three-dimensional angle between the femur and the tibia for the knee and the angle between the tibia and the foot for the ankle. Original data reported in [35,38].

Felids are one such group in which posture does not vary across body masses ranging from 4 to nearly 200 kg (Fig 3) [35]. Rather than becoming upright, felids maintain a crouched posture, even at large body sizes; this may enable the large horizontal ground reaction forces necessary for accelerating and manoeuvering [36], which, like speed, can increase fitness [37]. Varanid lizards are another group that shows a reduced effect of size on locomotor posture; they do not adopt a more upright posture as they increase in size from 0.04 to 8 kg, but rather they maintain a sprawled posture in which their femur is highly abducted in a push up-like position. This is functionally similar to the crouched posture of felids, and, like flexion, abduction reduces the mechanical advantage resisting the gravitational force (Fig 3) [38]. Studies on iguanids and alligators provide evidence that the lack of upright posture in larger varanids can be explained by the decrease in EMA and increase in musculoskeletal stresses, predominately due to increased torsional bone loading, that result when a sprawling animal transitions to an upright stance [9,39]. Yet despite this inability to become upright, varanid lizards still reach large body masses.

For example, the world’s largest lizard, the Komodo dragon, seems to defy the principles of animal posture and size, reaching masses of over 100 kg while still adopting an extremely sprawling and crouched posture. Geometric scaling would suggest stresses placed on the bones and muscles of these giant lizards would put them near failure points, causing fractures or tears during daily locomotor tasks. Yet Komodo Dragons seem to do quite a good job of getting around, reaching speeds of nearly 20 km h^-1^ [40] and managing to keep their musculoskeletal systems intact. So the question is how do large crouched animals such as lizards and felids deal with the size-related increases in stress? One possible mechanism could be to change the design of their skeletal muscles.

## Muscle Architecture Changes

The architecture of skeletal muscle in larger animals may be adapted to support the increase in mass. Skeletal muscle functions to change length and produce force, providing a role in both support and locomotion. All things being equal, larger muscles (either in mass or area) are capable of supporting or producing more force. Yet the arrangement of fibres within a muscle also affects its function. For example, pennate muscles are characterized by fibres that lie at an angle relative to its force-generating axis. This allows for (i) more muscle fibres to be packed within a given volume, increasing the physiological cross-sectional area, and (ii) muscle fibres to rotate to steeper pennation angles during contraction, thereby uncoupling fibre-shortening speed from movement speeds [41]. For larger animals, an increase in the relative thickness of a muscle (larger physiological cross-sectional area) would disperse forces over a greater area and lead to a decrease in muscle stress.

Some groups of animals do appear to alter the mechanical properties of their muscles to mitigate the size-related increases in stress. Mammals ranging in size from 0.009 to 2,500 kg increase the force-generating (or withstanding) capability of their muscles by increasing muscle mass or cross-sectional area, or by decreasing muscle fibre length [42,43]. Our work in varanids [12] and Cuff and colleagues’ work in felids [36,44] provide some of the first studies to determine how skeletal muscle architecture varies with size within groups in which posture is decoupled from body size. In lizards ranging from the 0.008 kg *Varanus brevicauda* to the 40 kg *Varanus komodoensis*, we found an increase in the relative force-generating capacity of femur adductors, knee flexors, and ankle plantarflexors, with positive allometry for either muscle mass or physiological cross-sectional area and, to a lesser extent, pennation angle (Fig 1). The lack of strong evidence for geometric changes suggests that larger animals keep the same relative shortening speed and use the extra muscle mass to increase the relative force-generating capacity of their muscles. This shift in skeletal muscle design towards an increased role in support is one possible explanation for how the Komodo dragon can reach such large body masses while still maintaining a sprawling posture.

In felids, the scaling trends were not as prominent. Only 1 of 38 hindlimb muscles scaled with positive allometry for muscle mass, suggesting that larger felids may simply suffer from weaker hindlimb muscles. The differences in scaling of muscle properties from our work in lizards [12] and from others in felids [44] can potentially be explained by the difference in posture between these two groups (Fig 3). Felids maintain a crouched parasagittal posture, whereas lizards are crouched and sprawling. A comparison of hindlimb joint angles within felids and lizards [35,38] shows that varanids have a higher degree of “crouchedness,” which may suggest that felids may not have to compensate as much as varanids. To overcome size-related increases in stress, large crouched animals either increase the size of their muscles (mass or area) to favour support, as shown in varanids, or simply deal with relatively weaker muscles, as shown in felids, both of which likely result in a decrease in locomotor performance.

## Speeds Get Slower

**Fig 4:**
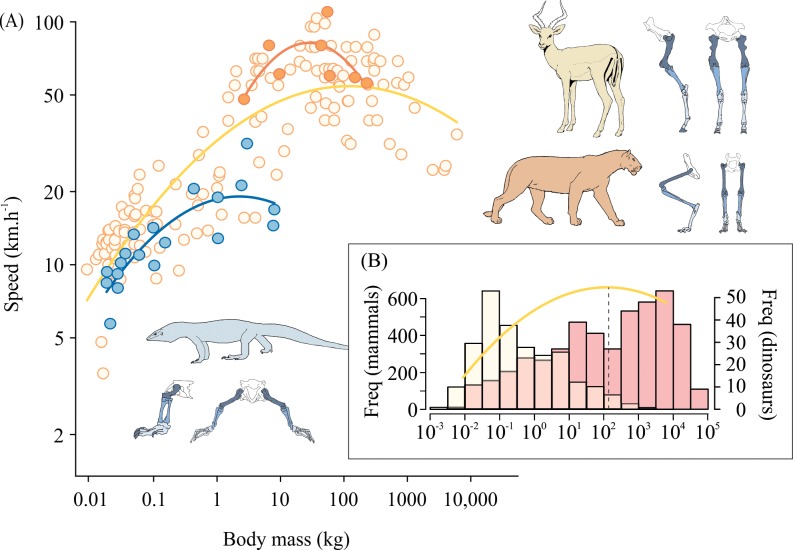
(A) Top running speeds are reached at different body sizes in (nonfelid) mammals (*n* = 142), felids (*n* = 8), and varanids (*n* = 19). The curvilinear relationship between body mass (kg) and running speed (km h^-1^) for mammals (yellow), felids (orange), and varanids (blue). Optimum body mass for speed is denoted by the peak of each curvilinear relationship. Original data reported in [40,46–51]. (B) Frequency distribution for the body masses of extant running mammals [52] (*n* = 2,919; yellow bars) and extinct dinosaur species [1] (*n* = 406; red bars) shown with the relationship of optimum mass for speed in nonfelid mammals.

There is no one universal strategy to reduce size-related increases in stress. Different groups of animals appear to use different biomechanical strategies to reduce the stresses acting on their musculoskeletal system. Even within closely related groups, the ability for these techniques to decrease stress over a large size range may be limited. Once this limit is reached, the only way to effectively reduce stress is to decrease locomotor speed. By slowing down, animals can distribute force over a longer ground contact time and effectively reduce peak stress [45]. When observing the relationship between speed and size, we see an initial increase in speed with body mass, owing to the increase in stride length of which larger animals are capable. But at increasingly larger sizes, stresses likely build up to a point at which stress reduction strategies are no longer effective. Above this body mass, we would expect speed to decrease in order to alleviate the stress, and this is indeed the case (Fig 4). But the mass at which speed starts to decrease may be based upon which stress reduction strategies are exploited—this may be relevant for estimating speeds of our extinct giants

Fig 4 shows the relationship between body mass and running speed for three groups of animals in which we have complete data sets examining different strategies that may be used to decrease musculoskeletal stress. Among varanids, we see changes in bones [28] and muscles [12], but owing to their sprawling posture, they are likely unable to become upright, and thus the mass at which speed starts to decline occurs at a low body size [49]. Mammals appear to use the greatest number of strategies to mitigate size-predicted increases in stress; some (i.e., ungulates) show changes in bone [21,22], muscle [42,43], and posture [31,32], becoming more upright as body size increases. The combined effect of these factors likely results in the ability for mammals to reach large body masses before the size-related increases in stress force them to move at slower speeds. Felids appear to be an intermediate between these two groups: their bones do appear to change [10,11], but their muscles do not [36,44]; they are parasagittal (but crouched), and their posture does not change with body mass [35]. Felids contain some of the fastest running animals, which may be selected for manoeuverability, but mechanisms for reducing stress are not as prominent as in other mammals, which probably results in the lower mass at which they slow down.

Body mass is the most obvious and arguably the most fundamental characteristic of an organism, impacting many important attributes of its life history, ecology, and evolution. One consistent and intriguing observation is that we see so few species above the optimum body mass for speed (Fig 4A). Specifically, among the nonfelid mammals, the optimum body mass with respect to speed was 121 kg, and only 18% of nonfelid mammals were above this mass (Fig 4B). Additionally, this number may be exaggerated by the greater proportion of large-bodied mammals for which running speeds are reported in the literature. In fact, when we observe the body mass distribution for a more comprehensive dataset of running mammals (*n* = 2,919; [52]), the body masses are heavily skewed towards a small body size (Fig 4B). Using this same optimum suggests that a very low percentage of extant mammal species (3.8%) are above the optimum mass for speed, with only slightly more (8%) if we include body size estimates from extinct mammals [52]. In varanids there is a similar pattern—only 21% of species are above the optimum, yet when we include additional body masses from varanids (*n* = 45; [53]) and other lizard species (*n* = 185; [54,55]), there remain even fewer species above the optimum mass for speed: 5.4% (all lizards). This may suggest that large slow species do not currently succeed in nature. So if in fact dinosaurs were using the same stress reduction strategies as mammals, we may expect a similar fraction of species above the optimum mass for speed. But the patterns we see amongst these extinct giants are different (Fig 4B). Using dinosaur body mass estimates [1], we observe that a much greater fraction of dinosaurs fall above this optimum—81% show body sizes larger than the optimum mass for speed predicted for mammals. Even when accounting for the reduced preservation for many small-bodied dinosaur species, the number of dinosaurs above the optimum is still higher than we might predict from patterns among extant lineages.

So then what allowed these animals to become so giant? Two possible solutions may explain the absence of giants among extant species. Either the combined effects from environmental, ecological, and biological factors (e.g., [56]) have drastically changed (or continue to change too quickly) such that they no longer allow massive slow animals to be successful in nature or, alternatively, dinosaurs evolved another stress reduction strategy that we do not currently see in extant species, pushing the optimum mass in relation to speed to a larger body size. Future efforts to reconstruct the locomotor patterns of these giant extinct animals using biomechanical modelling and computer simulations rigorously compared against experimental data from extant animals (e.g., [57,58]) is likely the most promising approach to help understand where all the giants have gone. Unless new strategies evolve to circumvent these size-predicted increases in stress or taxa are able to deal with the potentially deleterious effects of slow speed, we may never see the evolution of giants again.

## Abbreviations

EMA: effective mechanical advantage
